# Synchronous Acute Appendicitis and Acute Cholecystitis: A Report of a Rare Case

**DOI:** 10.7759/cureus.98850

**Published:** 2025-12-09

**Authors:** Samuel C Kim, Salvatore Grasso, Anthony McCloud, Gudata Hinika

**Affiliations:** 1 General Surgery, California Hospital Medical Center, Los Angeles, USA; 2 Medicine, Ross University School of Medicine, Los Angeles, USA

**Keywords:** appendicitis, cholecystitis, laparoscopy surgery, surgery, synchronous presentation

## Abstract

Two of the most common emergent surgical interventions are acute appendicitis and acute cholecystitis. However, synchronous presentation is rare. In this report, we present a 29-year-old female patient with three days of worsening right lower quadrant (RLQ) pain with nausea and vomiting. Physical examination showed tenderness to palpation at the RLQ. Workup showed acute appendicitis with probable localized perforation and peri-appendiceal abscess, and CT-guided drainage was performed. The patient was discharged after four days. Seventeen days later, the patient returned to the ED with complaints of new right upper quadrant (RUQ) pain with nausea, vomiting, and loss of appetite. The ultrasound showed similar gallbladder findings with a positive sonographic Murphy’s sign. A CT scan showed appendicitis and cholecystitis with gallstones and mild gallbladder wall thickening. The patient then had a laparoscopic cholecystectomy and appendectomy. This case report sheds new light on this rare and interesting surgical emergency.

## Introduction

Acute appendicitis and acute cholecystitis are very common in the United States. There are approximately 350,000 cases of appendicitis and 200,000 cases of cholecystitis annually [1,2]. Acute appendicitis often presents as pain in the periumbilical region that migrates to the right lower quadrant (RLQ) to McBurney’s Point, while acute cholecystitis often presents as pain in the right upper quadrant (RUQ) or epigastrium with possible radiation to the right shoulder or back and a positive Murphy’s sign. Both conditions often present with fever, nausea, vomiting, and/or anorexia.

Acute appendicitis is usually diagnosed by a CT scan, a medical imaging technique used to obtain detailed internal images of the body, and a physical examination. Sensitivity is reported at 0.95 (95% CI [0.93, 0.96]) and specificity at 0.94 (95% CI (0.92, 0.95)) using a CT scan to diagnose acute appendicitis [3]. Acute cholecystitis is usually diagnosed with an ultrasound, an imaging test that uses sound waves to make pictures of organs, tissues, and other structures inside the body, but a CT scan can also be effective. The ultrasound has the best unadjusted sensitivity (0.97; 95% CI (0.95, 0.99)) and specificity (0.95; 95% CI (0.88, 1.00)) for evaluating patients with acute cholecystitis [4].

Each individual condition and presentation in patients can be treated medically with antibiotics and symptom management or surgically with laparoscopic resection or open resection [5,6]. Laparoscopic surgery is the gold standard for both conditions, respectively [1,7]. There have been rare cases of synchronous appendicitis and cholecystitis. One literature review found only 11 case reports of coexistent acute appendicitis and acute cholecystitis [8]. Furthermore, one coexistent case of ruptured appendix and acute cholecystitis has been reported [9], while another case with ruptured gallbladder and acute appendicitis has been reported [10]. In this report, we present a unique, and to the best of our knowledge, first-of-its-kind case of acute appendicitis with appendiceal abscess with synchronous acute cholecystitis. 

This article was previously presented as a poster presentation at the 2024 California Hospital Medical Center (CHMC)/Ross University Medical School Annual Conference on May 31st, 2024.

## Case presentation

A 29-year-old Hispanic female patient presented to the emergency department with three days of worsening RLQ pain with associated nausea, vomiting, dysuria, and diarrhea. On the third day of pain, she went to see her primary medical doctor, who sent her to the emergency department (ED) to rule out acute appendicitis. She reported experiencing a similar episode one week earlier that had resolved spontaneously. Her only past medical history consisted of a prior cesarean section; she denied other medical, family, or social risk factors.

In the ED, the patient was afebrile (36.4°C) with a heart rate of 85 bpm, respiratory rate of 20, blood pressure of 132/86 mmHg, and oxygen saturation of 97% on room air. Her abdomen was mildly distended and exhibited marked RLQ tenderness to even light palpation, accompanied by guarding. There was no rigidity, no rebound tenderness, and both the obturator and Rovsing’s signs were negative. Laboratory studies (Table 1) revealed leukocytosis to 18.9 thousand with a left shift (87% neutrophils) and otherwise unremarkable electrolytes and liver function tests.

**Table 1:** Laboratory values on presentation and following presentation

Parameters	Initial lab value	Lab values following presentation	Reference range
WBC	18.9	8.2	4,500–11,000/mm3
RBC	4.64	4.81	4.40-6.00 M/uL
Hemoglobin	13.1	13.6	12.0-16.0 g/dL
Hematocrit	38.4	39.4	36%-46%
Mean corpuscular volume	82.6	81.9	80-100 fL
Mean corpuscular hemoglobin	28.3	28.2	25.0-35.0 pg
Mean corpuscular hemoglobin concentration	34.2	34.4	31%-36% Hb/cell
Red cell distribution width	13.1	13.2	<16.4 %
Platelet	385	381	150,000-400,000/mm3
Mean platelet volume	6.8	7.2	7-9 fL
Absolute neutrophil count	16.4	5.1	2.0-8.0 K/uL
ABS lymphocyte count	1.3	2.5	1.0-5.1 K/uL
ABS monocyte count	1.1	0.4	0.0-0.8 K/uL
ABS eosinophil count	0.0	0.2	0.0-0.5 K/uL
ABS basophil count	0.0	0.0	0.0-0.2 K/uL
Neutrophil %	87.0	62.2	49.0-74.0 %
Lymphocyte %	6.9	30.3	26.0-46.0 %
Monocyte %	5.8	4.9	2.0-12.0 %
Eosinophil %	0.1	2.0	0.0-5.0 %
Basophil %	0.2	0.6	0.0-2.0 %
Sodium	136	137	136-146 mEq/L
Potassium	4.1	5.1	3.5-5.0 mEq/L
Chloride	100	106	95-105 mEq/L
CO2	24	20	22-28 mEq/L
Anion gap	12	11	8-16 mEq/L
Glucose	114	120	<140 mg/dL
Blood urea nitrogen (BUN)	8.00	11.0	7-18 mg/dL
Creatinine	0.8	0.8	0.6-1.2 mg/dL
BUN/creatinine ratio	10.1	14.5	10-20:1
Estimated glomerular filtration rate (eGFRcr)	104	109	90-120 mL/min/1.73m
Calcium	9.4	9.1	8.4-10.2 mg/DL
Protein, total	8.60	8.40	6.0-7.8 g/dL
Albumin (A)	4.0	3.9	3.5-5.5 g/dL
Globulin (G)	4.6	3.9	2.3-3.5 g/dL
A/G ratio	0.9	4.5	1.1-2.5
Bilirubin total	0.5	0.2	0.1-1.0 mg/dL
Alanine transaminase (ALT)	30	28	10-40 U/L
Aspartate aminotransferase (AST)	23	33	12-39 U/L
Alkphos	92.0	71.0	25-100 U/L
Lipase	9	25	13-60 U/L
Urianalysis: Color	Yellow	Straw	-
Appearance	Hazy	Clear	-
Specific gravity	1.026	1.025	1.005-1.030
Urinary pH	7.5	5.5	4.5-8
Urinary glucose	Negative	Negative	Negative
Urinary bilirubin	Negative	Negative	Negative
Urinary ketones	10 mg/dL	Negative	Negative
Urinary blood	Moderate	Small	Negative
Urinary protein	50 mg/dL	Negative	Negative
Urianalysis urobilinogen	Negative	Negative	Negative
Urinary nitrite	Negative	Negative	Negative
Urinary leukocyte esterase	Small	Moderate	Negative
Urinary analysis squamous epithelial	17/HPF	21/HPF	Negative
Urine WBCs	6-10	3-5	Negative
Urinary RBC	16-25	6-10	Negative
Urinary mucous	Rare	Rare	Negative
Urinary bacteria	Rare	Rare	Negative

The patient's urinalysis demonstrated pyuria suggestive of a mild urinary tract infection. Given the physical exam findings, the ED physicians opted to bypass ultrasound and proceed with CT imaging for further evaluation. A CT abdomen and pelvis with IV contrast showed findings consistent with acute appendicitis with probable localized perforation and peri-appendiceal abscess. The patient received Toradol, Zosyn, and 1 L of normal saline (NS) bolus x two before admission to the floor.

The following day, the patient underwent CT-guided IR drainage of the abscess. A right lower quadrant drain was placed, and purulent material was noted upon insertion. She continued to improve clinically and was discharged on postoperative day four with strict return precautions and instructions for outpatient drain monitoring and eventual removal.

**Figure 1:**
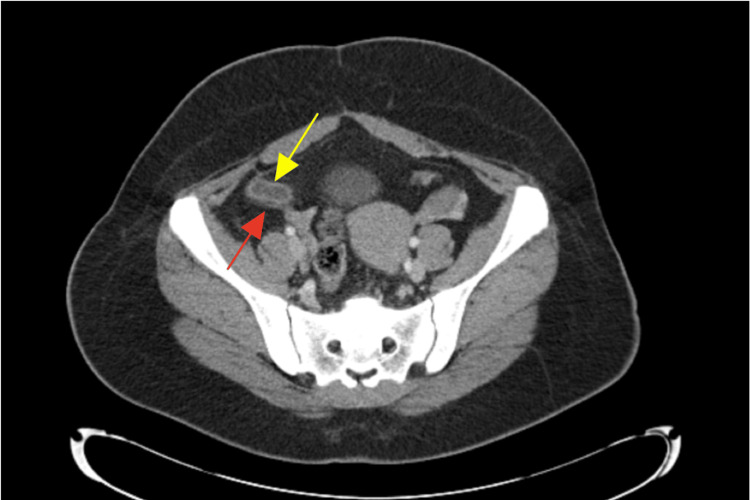
Transverse CT of abdomen on postoperative day eight Yellow arrow: Appendiceal wall thickening without an overt source of obstruction; Red arrow: Fat stranding surrounding the appendix signifying an inflammatory reaction

On postoperative day eight, the patient returned to the ED requesting removal of the drain. She reported only mild discomfort and approximately 15 cc of output in the accordion reservoir. Repeat CT imaging demonstrated no residual abscess but persistent inflammation surrounding the appendix (Figure 1). Given these findings, the decision was made to leave the drain in place for an additional week, and she was discharged home.

Five days later, the patient again presented to the ED with mild abdominal pain and continued minimal drainage from the catheter. Because she had a history of a perforated appendicitis with ongoing drainage, repeat imaging was clinically justified. The CT imaging revealed a fluid-filled, distended appendix unchanged from prior studies and no remaining fluid collections. New findings included gallbladder sludge and stones. As the abscess had resolved and the drain was no longer necessary, it was removed, and the patient was discharged in stable condition.

Approximately 20 days after her initial appendicitis and abscess drainage, the patient returned to the ED with a two-day history of worsening RUQ pain accompanied by mild residual RLQ discomfort. She reported that the pain typically began about 10 minutes after eating and radiated to her mid-back, noting that she had experienced similar episodes in the past. She also endorsed associated nausea and decreased appetite, but denied any vomiting.

**Figure 2:**
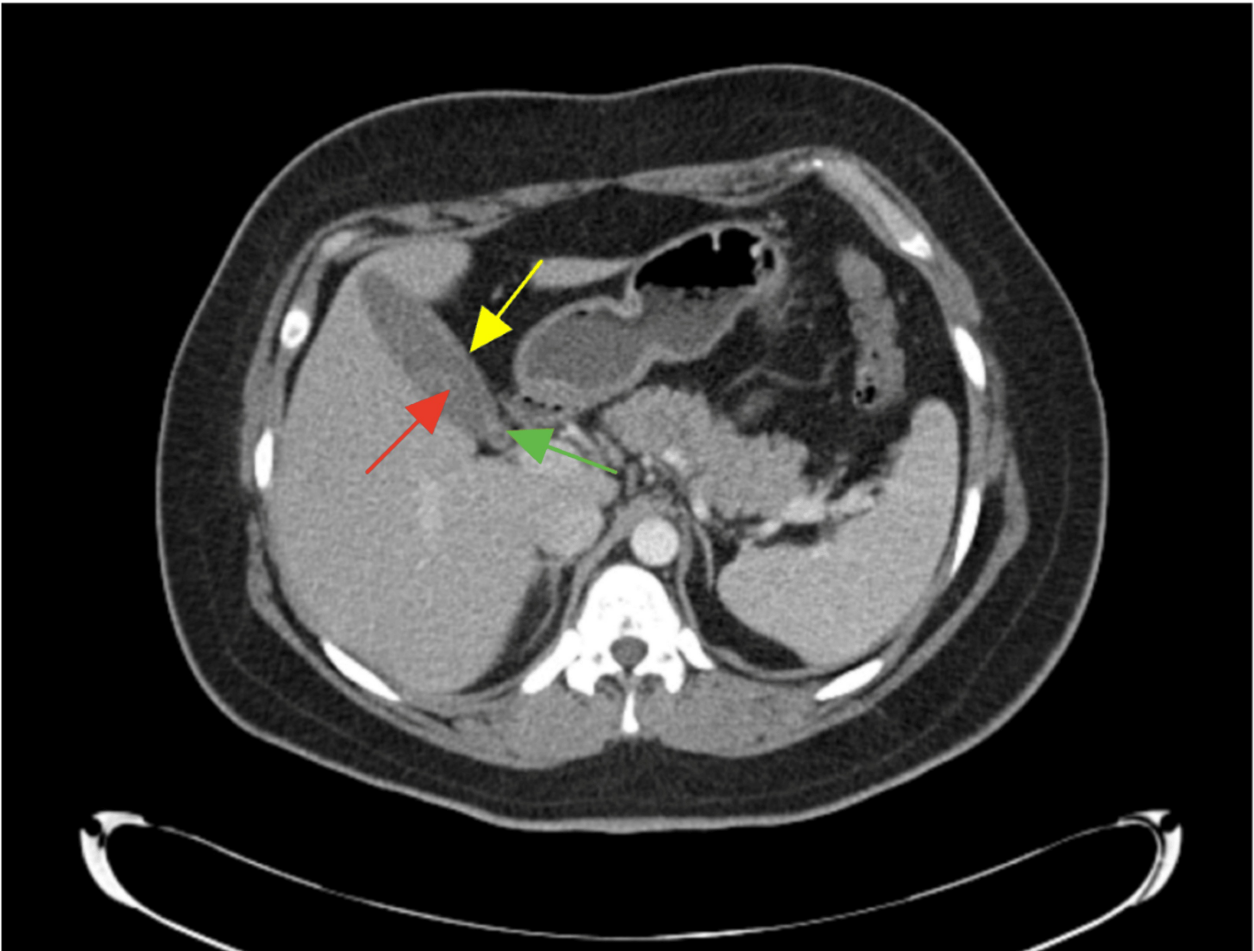
Transverse CT of abdomen on postoperative day 20 Yellow arrow: Mild gallbladder wall thickening; Red arrow: Presence of gallstones; Green arrow: Gallstone evident at the neck of the gallbladder as a possible source of obstruction to outflow of bile secretion

On examination, the patient was afebrile at 36.6°C with a heart rate of 63 bpm, respiratory rate of 18, blood pressure of 109/74 mmHg, and oxygen saturation of 97% on room air. She exhibited focal tenderness to palpation in the RUQ. Laboratory evaluation revealed a WBC count of 8.2 × 10³/µL with 62.2% neutrophils and otherwise normal complete blood count (CBC) and comprehensive metabolic panel (CMP) values (see Table 1). Urinalysis was notable for asymptomatic bacteriuria. The CT abdomen and pelvis (Figure 2) with IV contrast showed a fluid-filled appendix with mucosal thickening and surrounding inflammation changes consistent with acute appendicitis, though less dilated than prior CT, with gallstones and gallbladder wall thickening. The RUQ ultrasound showed gallstones with wall thickening and a positive sonographic Murphy’s sign. General surgery was consulted, and a combined laparoscopic cholecystectomy and appendectomy was planned.

Preoperatively, the patient was resuscitated with IV fluids. She received IV pain control and was started on empiric IV antibiotics with Zosyn (piperacillin-tazobactam). The following day, she was taken to the operating room for a laparoscopic cholecystectomy and appendectomy, which she tolerated well without complications. Postoperatively, she resumed a regular diet and had a return of bowel function, allowing for discharge on postoperative day four with a prescribed course of antibiotics. Her recovery was uneventful.

## Discussion

Classical presentations of both pathologies are extensively described in medical literature, but their synchronous presentation varies for every patient, which may lead to challenges in diagnosis and management. In our unique patient, her initial appendiceal abscess with appendicitis was treated medically, as that was deemed safer for the patient [11]. After her synchronous development of acute cholecystitis with appendicitis, medical management was no longer safe for the patient, and urgent surgical intervention was planned. 

Carter et al. [12] described biliary reflux or gallbladder dyskinesia associated with acute appendicitis that was relieved after an appendectomy. Another report suggested that the direct bacterial invasion or translocation from the muscularis propria of a gangrenous appendix into the portal venous system caused impairment of bile salt excretion, leading to acute cholecystitis, which was managed surgically [8]. The Kancheva et al. [13] case report of synchronous acute appendicitis and acute cholecystitis summarized some synchronous cases and showed that all but one case were treated surgically. The gold standard of permanent treatment for acute cholecystitis remains surgical resection [14]. Though the Antibiotic Therapy vs Appendectomy for Treatment of Uncomplicated Acute Appendicitis (APPAC) [15] trials established that antibiotic medical treatment of uncomplicated appendicitis is appropriate, our patient’s case of appendicitis was complicated. Buhamed et al. [8] report that surgical management in synchronous cases is appropriate to avoid complications of perforation or septicemia.

With few reported cases of simultaneous pathologies, with possibly no prior reported cases of an appendiceal abscess with appendicitis and synchronous acute cholecystitis, it is difficult to explain the etiology. Drawing from the literature review, which reports a 73.6% rate [16] favoring operative intervention, we find that surgical management offers superior outcomes for patients with synchronous appendicitis and cholecystitis compared with medical management. Therefore, a five-port laparoscopic approach is recommended as an appropriate and effective operative strategy.

## Conclusions

Concurrent acute appendicitis and acute cholecystitis remain a medical mystery and a surgical urgency. Thorough history, physical examination, and imaging workup are essential for diagnosis. Surgical interventions, especially laparoscopic procedures, remain the gold standard. This case underscores the importance of recognizing and managing this rare condition.
